# ﻿Taxonomy of the genus *Elasmopus* (Crustacea, Amphipoda) in Japan and South Korea, with description of a new species

**DOI:** 10.3897/zookeys.1261.162923

**Published:** 2025-12-02

**Authors:** Hiro Yoshimura, Chi-Woo Lee, Ko Tomikawa

**Affiliations:** 1 Graduate School of Humanities and Social Sciences, Hiroshima University, 1-1-1 Kagamiyama, Higashihiroshima, Hiroshima 739-8524, Japan Hiroshima University Hiroshima Japan; 2 Nakdonggang National Institute of Biological Resources, Sangju 37242, Republic of Korea Nakdonggang National Institute of Biological Resources Sangju Republic of Korea

**Keywords:** COI, East Asia, genetic distance, Kimura two-parameter distance, morphology, *p*-distance

## Abstract

To advance the limited understanding of the East Asian *Elasmopus* fauna, field surveys were conducted in Japan and South Korea. In the present study, a new species, *E. lumbiniger***sp. nov.**, is described. The new species is distinguished from its congeners by a strongly projected posteroventral corner of the epimeral plate 3, long slender setae on the anterodistal corner of the male gnathopod 2 carpus, a mid-palmar ridge on the male gnathopod 2 propodus, and a smooth posterior margin on the basis of pereopods 5–7. In addition, *E. koreanus* is recorded in Japan for the first time, and the known distribution range of *E. mukuinu* has been significantly extended. The nucleotide sequences of the mitochondrial cytochrome *c* oxidase subunit I (COI) of these species were determined, and the genetic distances among *Elasmopus* species were provided. A key to the species of *Elasmopus* found in East Asia is also provided.

## ﻿Introduction

The genus *Elasmopus* Costa, 1853 is distributed in the shallow waters of tropical and temperate regions worldwide (Lowry and Hughes 2009; Vader and Krapp-Schickel 2012). To date, 124 *Elasmopus* species have been described (Vader and Krapp-Schickel 2012; Alves et al. 2016; Horton et al. 2025; Yoshimura and Tomikawa 2025). Of these, 13 species of *Elasmopus* are currently known from East Asia (Table 1).

**Table 1.** Occurrence records of *Elasmopus* species in East Asia.

Species	Occurrence in East Asia	Notes	References
*E. alkhiranensis* Myers & Momtazi, 2015	South China Sea, China	Ren (2012) recorded this species as *E. pectenicrus* (Bate, 1862); however, it was referred to *E. alkhiranensis* by Myers and Momtazi (2015).	Ren (2012); Myers and Momtazi (2015)
*E. hawaiensis* Schellenberg, 1938	South China Sea, China	-	Ren (2012)
*E. hooheno* Barnard, 1970	South China Sea, China	-	Ren (2012)
*E. japonicus* Stephensen, 1932	Philippine Sea, Japan	-	Stephensen (1932)
*E. koreanus* Kim & Kim, 1991	Sea of Japan (East Sea), South Korea, and Japan; Pacific Ocean, Japan	-	Kim and Kim (1991); Kim et al. (2023); Shin et al. (2025); the present study
*E. lumbiniger* sp. nov.	Sea of Japan, Japan (East Sea); Philippine Sea, Japan; Pacific Ocean, Japan	-	present study
*E. mukuinu* Sir & White, 2022	Philippine Sea, Japan	-	Sir and White (2022); present study
*E. nanshaensis* Ren, 1998	South China Sea, China	-	Ren (2012)
*E. nkjaf* Nakamura, Nakano, Ota & Tomikawa, 2019	Philippine Sea, Japan	-	Nakamura et al. (2019)
*E. projectus* Yoshimura & Tomikawa, 2025	Sea of Japan (East Sea), Japan; Pacific Ocean, Japan	-	Yoshimura and Tomikawa (2025)
*E. rapax* Costa, 1853	South China Sea, China; Sea of Japan (East Sea), South Korea	-	Shin et al. (2005); Ren (2012); Shin et al. (2025)
*E. spinicarpus* Berents, 1983	South China Sea, China	-	Ren (2012)
*E. spinidactylus* Chevreux, 1907	South China Sea, China	-	Ren (2012)
*E. spinimanus* Walker, 1904	South China Sea, China	-	Ren (2012)

In Japan, *E. japonicus* and *E. projectus* have been recorded from temperate coastal areas, and *E. mukuinu* and *E. nkjaf* from subtropical zones (Stephensen 1932; Nakamura et al. 2019; Sir and White 2022; Yoshimura and Tomikawa 2025). Two species, *E. koreanus* and *E. rapax*, have been recorded from South Korean waters (Kim and Kim 1991; Shin et al. 2005; Kim et al. 2023; Shin et al. 2025). However, many regions in Japan and South Korea remain taxonomically unstudied with regard to *Elasmopus*. The presence of several unrecorded or undescribed species of *Elasmopus* has been suggested (Hirayama 1995; Ariyama 2022).

To clarify the *Elasmopus* fauna in Japan and South Korea, we conducted field surveys at multiple sites. We found *E. koreanus* and *E. mukuinu* in temperate regions of Japan, where they had not been previously recorded, as well as an undescribed species, *E. lumbiniger* sp. nov. Additionally, we sequenced the mitochondrial cytochrome *c* oxidase subunit I (COI) gene of the collected samples and calculated the genetic distances among the *Elasmopus* species.

## ﻿Materials and methods

### ﻿Field sampling

**Figure 1.**
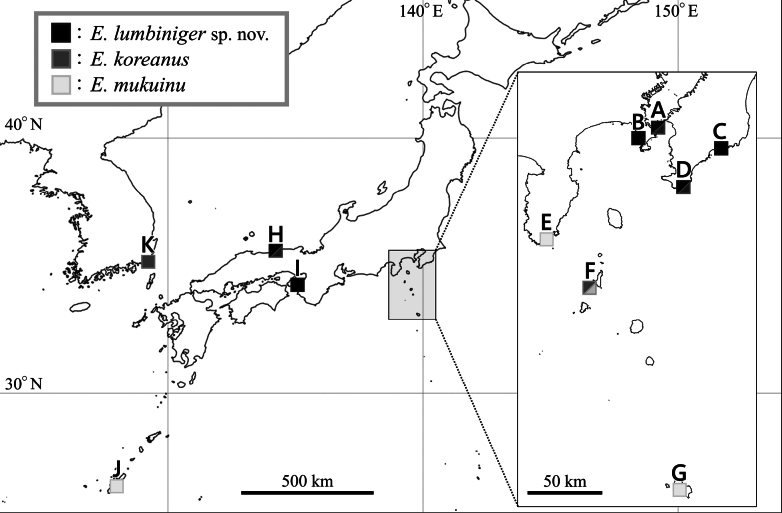
Map showing collecting sites of the examined specimens. Slashes within boxes indicate that two species were collected from the same site. **A.** Kannonzaki Coast, Kanagawa, the type locality of *Elasmopus lumbiniger* sp. nov.; **B.** Arasaki Coast, Kanagawa; **C.** Uchiura Beach, Chiba; **D.** Shirahama, Chiba; **E.** Ono Beach, Shizuoka; **F.** Ôura Beach, Shikinejima Island, Tokyo; **G.** Yakengahama Beach, Hachijojima Island, Tokyo; **H.** Uradome Beach, Tottori; **I.** Takurazaki, Wakayama; **J.** Kaichu Doro, Okinawa Island, Okinawa, the type locality of *E. mukuinu*; **K.** Songjeonggudeokpo-gil, Busan.

The specimens were collected from the intertidal and shallow subtidal zones during low tide, depth range 0–1 m. The sampling sites are shown in Fig. 1. While still alive, some specimens were photographed using a digital camera (EOS M6; Canon, Tokyo, Japan) with a lens (LAOWA 65 mm F2.8 Macro Photo Lens; Venus Optics, Heifei, China). After photographs were taken, some specimens were fixed and preserved in 99% ethanol while others were frozen, fixed, and preserved in propylene glycol.

### ﻿Morphological examinations

All appendages were dissected in 50% ethanol and mounted in gum-chloral medium on glass slides under a stereomicroscope (SZ61; Olympus, Tokyo, Japan). The specimens were examined using a light microscope (Eclipse Ni-U; Nikon, Tokyo, Japan) and illustrated with the aid of a camera lucida (Y-IDT Drawing Tube; Nikon, Tokyo, Japan). Body length, measured from the rostrum tip to the base of the telson along the dorsal curvature, was recorded to the nearest 0.1 mm. Some slide preparations were photographed using a device that integrated a digital camera (EOS M6) with an imaging lens (EF-M 55-200 mm F4.5–6.3 IS STM; Canon, Tokyo, Japan) and an objective lens (UPlan Fl 10x/0.30; Olympus, Tokyo, Japan). The examined specimens are deposited in the National Museum of Nature and Science, Tsukuba (**NSMT**), and Nakdonggang National Institute of Biological Resources (**NNIBR**).

### ﻿DNA sequencing

Genomic DNA was extracted from the pereonites and pleonites muscles of the specimens, using the protocol described by Tomikawa et al. (2014). The primer set used for polymerase chain reaction (PCR) and cycles sequencing (CS) was LCO1490 and HCO2198 (Folmer et al. 1994). PCR and CS mixtures were prepared following Tomikawa et al. (2014). PCR was performed as follows: enzyme activation for 5 min at 94 °C; 35 cycles of 30 s at 94 °C, 60 s at 50 °C, and 90 s at 72 °C; and final extension for 6 min at 72 °C. For samples with poor gene amplification efficiency under the above condition, the annealing temperature was adjusted from 50 to 42 °C. CS was performed as follows: 1 min at 96 °C; 25 cycles of 10 s at 96 °C, 5 s at 50 °C, and 4 min at 60 °C. The obtained sequences were assembled and edited using MEGA 11 (Tamura et al. 2021). The DNA sequence has been deposited with the International Nucleotide Sequence Database (INSD) through the DNA Data Bank of Japan (DDBJ). The INSD accession numbers are LC872568–LC872582 (Table 2).

**Table 2.** Specimen details and GenBank accession numbers used in the present study. Newly obtained sequences are indicated by an asterisk.

Species	Sample number	Locality	Coordinates (decimal degrees)	GenBank accession number
*E. koreanus*	NSMT-Cr 33060	Shirahama, Chiba, Japan	34.9031°N, 139.8862°E	*LC872568
	NSMT-Cr 33062	Uradome, Tottori, Japan	35.5915°N, 134.3220°E	*LC872569
	NNIBRIV137288	Songjeonggudeokpo-gil, Busan, South Korea	35.1682°N, 129.1975°E	*LC872570
*E. lumbiniger* sp. nov.	NSMT-Cr 33064	Kannonzaki Coast, Kanagawa, Japan	35.2592°N, 139.7436°E	*LC872571
	NSMT-Cr 33072	Arasaki Coast, Kanagawa, Japan	35.1947°N, 139.5996°E	*LC872572
	NSMT-Cr 33073	Uchiura Beach, Chiba, Japan	35.1262°N, 140.1893°E	*LC872573
	NSMT-Cr 33074	Shirahama, Chiba, Japan	34.9031°N, 139.8862°E	*LC872574
	NSMT-Cr 33076	Takurazaki, Wakayama, Japan	34.2671°N, 135.0605°E	*LC872575
	NSMT-Cr 33078	Uradome, Tottori, Japan	35.5921°N, 134.3198°E	*LC872576
*E. mukuinu*	NSMT-Cr 33079	Okinawa Island, Okinawa, Japan	26.3324°N, 127.9222°E	*LC872577
	NSMT-Cr 33080	Ono Beach, Shizuoka, Japan	34.6329°N, 138.8975°E	*LC872578
	NSMT-Cr 33082	Shikinejima Island, Tokyo, Japan	34.3307°N, 139.2083°E	*LC872579
	NSMT-Cr 33084	Hachijôjima Island, Tokyo, Japan	33.1007°N, 139.7696°E	*LC872580
*E. nkjaf*	KUZ Z1864	Miyako Island, Okinawa, Japan	24.7997°N, 125.3342°E	LC215812
	KUZ Z1862	Miyako Island, Okinawa, Japan	24.7997°N, 125.3342°E	LC215813
*E. projectus*	NSMT-Cr 32973	Kannonzaki Coast, Kanagawa, Japan	35.2592°N, 139.7436°E	LC851065
	NSMT-Cr 32974	Kannonzaki Coast, Kanagawa, Japan	35.2592°N, 139.7436°E	*LC872581
	NSMT-Cr 32980	Uradome, Tottori, Japan	35.5921°N, 134.3198°E	*LC872582
*E. rapax*	SFAM20-013	Somewhere along the west coast of Portugal	-	KF369125
	SFC18-003	Viana do Castelo, Minho, Portugal	41.70°N, 8.85°W	KX224027
	SFAM12-002	Viana do Castelo, Minho, Portugal	41.70°N, 8.85°W	KX224028
	SFAM20-001	Viana do Castelo, Minho, Portugal	41.70°N, 8.85°W	KX224029
	SFAM20-002	Viana do Castelo, Minho, Portugal	41.70°N, 8.85°W	KX224030
	SFAM20-003	Viana do Castelo, Minho, Portugal	41.70°N, 8.85°W	KX224031

### ﻿Genetic distances

Genetic distances within and among *E. koreanus*, *E. lumbiniger* sp. nov., *E. mukuinu*, *E. nkjaf*, *E. projectus*, and *E. rapax* were calculated. Nucleotide sequences of *E. nkjaf*, *E. projectus*, and *E. rapax* were obtained from NCBI database (https://www.ncbi.nlm.nih.gov/) (Table 2). Sequence alignment was performed using the MUSCLE algorithm implemented in MEGA 11. Genetic divergences were estimated based on uncorrected pairwise distance (*p*-distance) and Kimura’s two-parameter model (K2P) (Kimura 1980).

## ﻿Results

### ﻿Systematics

**Family Maeridae Krapp-Schickel, 2008**

**Genus *Elasmopus* Costa, 1853**

#### ﻿ Elasmopus koreanus

Taxon classificationAnimaliaAmphipodaMaeridae

Kim & Kim, 1991

F8431828-103B-53DD-B0F3-E36DBE786A72

**Figure 2.**
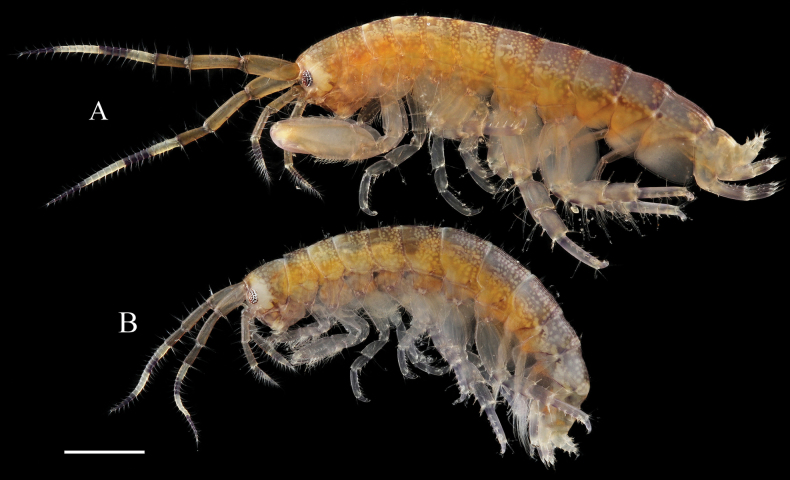
*Elasmopus koreanus*. **A.** Male, 6.4 mm (NSMT-Cr 33062), lateral view; **B.** Female, 6.2 mm (NSMT-Cr 33063), lateral view. Scale bar: 1.0 mm.

**Figure 3.**
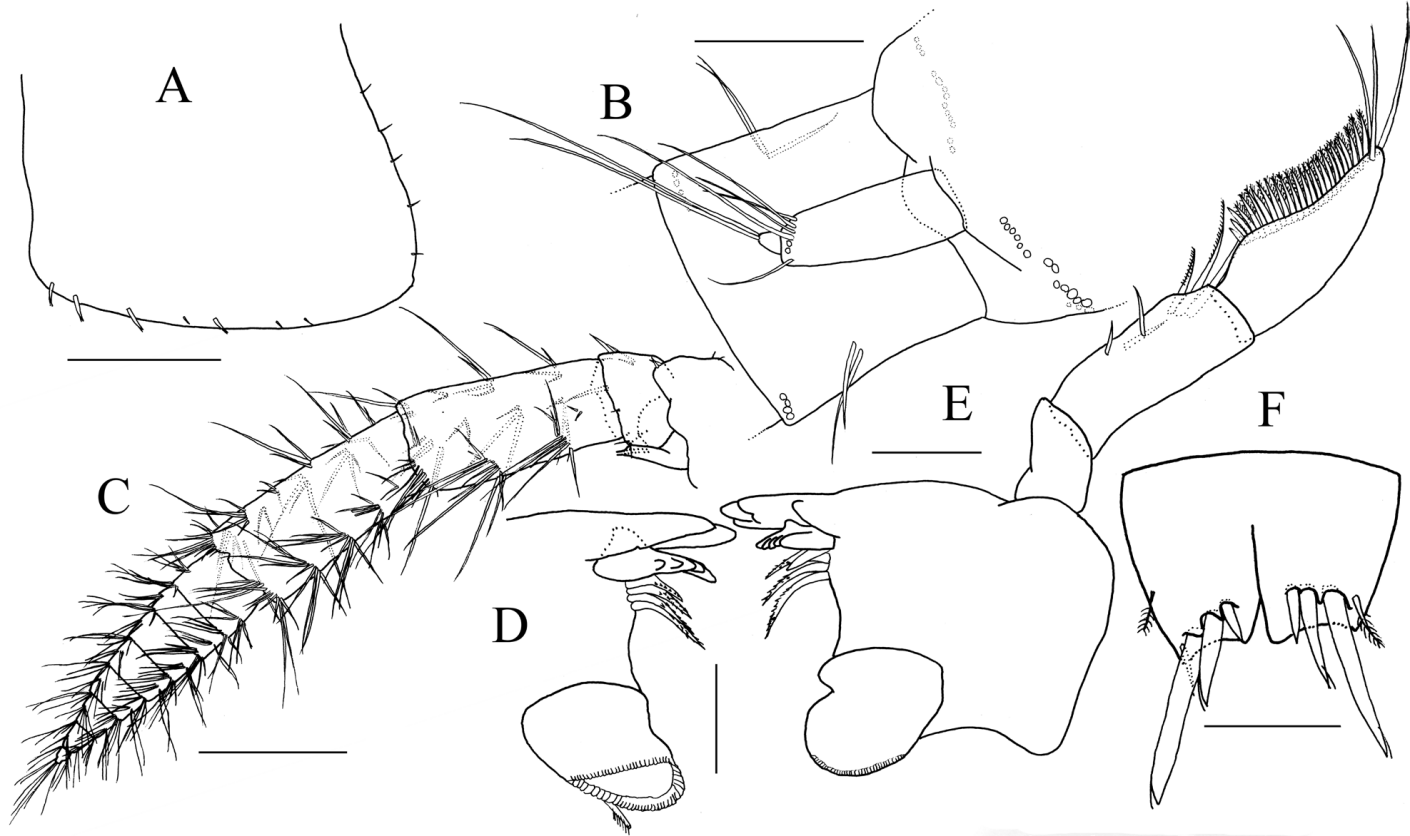
*Elasmopus koreanus*, male, 6.4 mm (NSMT-Cr 33062). **A.** Left epimeral plate 3, lateral view; **B.** Right accessory flagellum, medial view; **C.** Left antenna 2, dorsal view; **D.** Incisor, lacinia mobilis, accessory setal row and molar process of left mandible, medial view; **E.** Right mandible, medial view; **F.** Telson, dorsal view. Scale bars: 0.3 mm (**A, C**); 0.1 mm (**B, D–F**).

**Figure 4.**
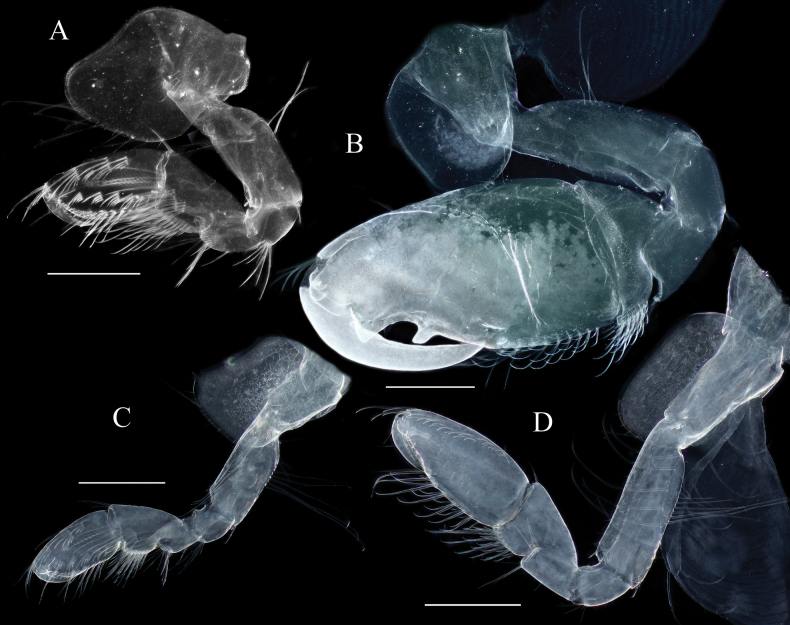
*Elasmopus koreanus*, **A, B.** Male, 6.4 mm (NSMT-Cr 33062); **C, D.** Female, 6.2 mm (NSMT-Cr 33063). **A.** Right gnathopod 1, medial view; **B.** Left gnathopod 2, lateral view; **C.** Right gnathopod 1, medial view; **D.** Right gnathopod 2, medial view. Scale bars: 0.3 mm.

**Figure 5.**
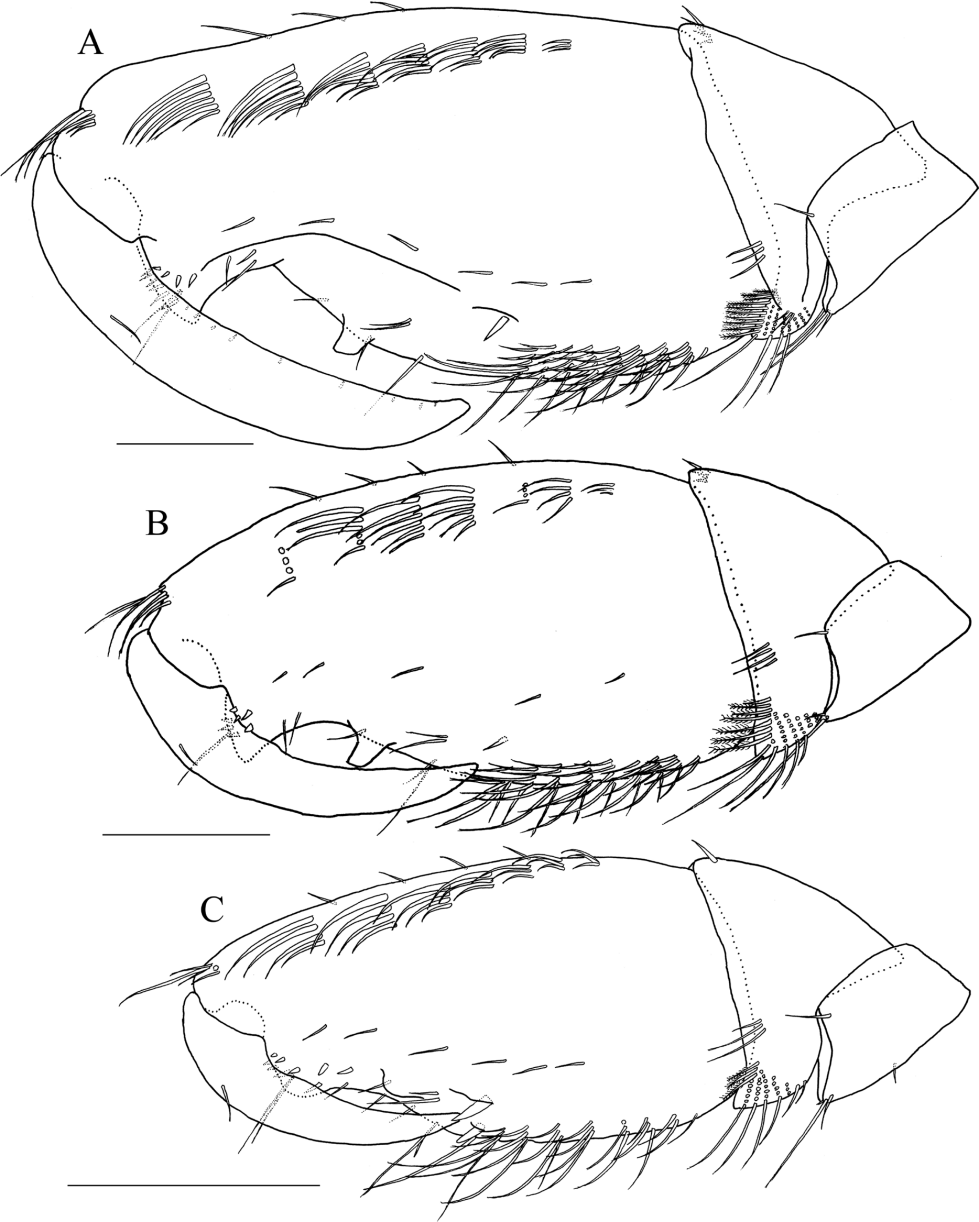
*Elasmopus koreanus*, ontogenetic morphological change in male gnathopod 2, merus to dactylus of right gnathopod 2, medial view. **A.** Male, 10.1 mm (NSMT-Cr 33059); **B.** Male, 6.4 mm (NSMT-Cr 33062); **C.** Male, 5.0 mm (NNIBRIV137288). Scale bars: 0.3 mm.

Figs 2, 3, 4, 5 [New Japanese name: Kourai-iso-yokoebi]

Elasmopus koreanus Kim & Kim, 1991: 329, figs 6, 7.

##### Material examined.

• Male, 10.1 mm (NSMT-Cr 33059), Kannonzaki Coast, Yokosuka, Kanagawa, Japan (35.2590°N, 139.7429°E) (Fig. 1A), shallow subtidal zone, rocky shore, among holdfast of the brown alga *Sargassum fusiforme*, 1 March 2022, H. Yoshimura coll.; • male, 8.3 mm (NSMT-Cr 33060), Shirahama, Minamiboso, Chiba, Japan (34.9031°N, 139.8862°E) (Fig. 1D), intertidal zone, rocky shore, 5 June 2023, H. Ogawa coll.; • male, 8.3 mm (NSMT-Cr 33061), Oura Beach, Shikinejima, Tokyo, Japan (34.3307°N, 139.2083°E) (Fig. 1F), shallow subtidal zone, rocky shore, among the red algae, 3 August 2024, H. Yoshimura coll.; • male, 6.4 mm (NSMT-Cr 33062: Fig. 2A), ovigerous female, 6.2 mm (NSMT-Cr 33063: Fig. 2B), Uradome Beach, Iwami, Tottori, Japan (35.5915°N, 134.3220°E) (Fig. 1H), shallow subtidal zone, rocky shore, among holdfast of the red alga *Hypnea* sp., 16 June 2024, H. Ogawa, Y. Mukaida, M. Ooga, and H. Yoshimura coll.; • male, 5.0 mm (NNIBRIV137288), Songjeonggudeokpo-gil, Haeundae-gu, Busan, South Korea (35.1682°N, 129.1975°E) (Fig. 1K), tide pool, rocky shore, among the brown alga *Sargassum thunbergii*, 26 September 2024, H. Yoshimura, C.W. Lee and K. Tomikawa coll.

##### Type locality.

Sadong, Ulleungdo Island, South Korea (37.472°N, 130.888°E; assumed from Kim and Kim 1991: fig. 1).

##### Diagnosis.

Epimeral plate 3 posteroventral corner without distinct projection. Antenna 1 peduncles bearing slender setae on lateral and medial margins, with bi-articulate accessory flagellum. Antenna 2 flagellum articles distinctly broader than long. Mandibular palp article 3 short. Male gnathopod 2 propodus posterior margin with a few clusters of slender setae, palmar margin with subtriangular process near hinge of dactylus, mid-palmar margin with subrectangular hump. Length of setae on coxae 2 and 3 shorter than width of each coxa. Pereopods 5–7 basis posterior margin smooth, without long setae. Telson cleft, border than long; lobe trapezoid, with shallow incision at apex, each lobe bearing one plumose and two or three robust setae apically.

##### Description of male

**(NSMT-Cr 33062). *Body*** (Fig. 2A): smooth, not carinate, with a few short setae dorsally on pereon and abdomen. ***Head***: eyes oval; lateral cephalic lobe broad, anteroventral margin with notch; dorsal surface with a few short setae. ***Epimeral plate 3*** (Fig. 3A): ventral margin with seven robust setae, posterior margin with five short setae, posteroventral corner without distinct projection.

***Antenna 1***: length 0.6 × body length; peduncular articles 1–3 with slender setae on lateral and medial margins; accessory flagellum (Fig. 3B) bi-articulate, reaching 2/3 of primary flagellar article 1, accessory flagellar article 2 tiny; primary flagellum 21-articulate, with slender setae. ***Antenna 2*** (Fig. 3C): length 0.4 × antenna 1; peduncular articles 4 and 5 almost same length, with slender setae on lateral and medial margins; flagellum 8-articulate, articles 1–5 shorter than wide, with slender setae.

***Mandible*** (Fig. 3D, E) with left and right incisors with two and four teeth, respectively; both left and right lacinia mobilis with five teeth; accessory setal row consisting of four setae on each of left and right mandibles; molar process well developed, triturative; palp well developed, tri-articulate; palp article 1 without setae, article 2 with six setae, article 3 falcate, length 2.5 × width.

***Gnathopod 1*** (Fig. 4A): subchelate; coxa anteroventral corner weakly produced, ventral margin with long and short setae; basis with long setae on posterior margin and medial surface; carpus subequal in length to propodus, with slender seta on anterodistal corner, with dense setae on posterior margin and medial surface; propodus with three and six clusters of setae on anterior margin and medial surface, respectively, posterior margin with row of slender and robust setae, palmar margin almost transverse, minutely serrate, with rows of robust setae on medial and lateral palmar submargins. ***Gnathopod 2*** (Figs 4B, 5B): subchelate; coxa with long and short setae on ventral margin, length of the longest seta 0.6 × width of coxa; basis with long setae on posterior margin; carpus with dense setae on posterior margin; propodus with four setae on anterolateral submargin, posterior margin with nine clusters of slender setae, palmar margin with subrectangular hump at midpoint and robust seta at proximal end where tip of dactylus contacts, lateral palmar margin with subtriangular hump near hinge of dactylus.

***Telson*** (Fig. 3F): broader than long, cleft for 60% of length; lobe trapezoid, with shallow incision at apex, each lobe bearing one plumose and three robust setae apically, lateral margin of each lobe with none or one plumose setae.

##### Description of female

**(NSMT-Cr 33063), sexually dimorphic characters. *Gnathopod 1*** (Fig. 4C): propodus anterior margin with one single and three clusters of setae, medial surface with five clusters of setae. ***Gnathopod 2*** (Fig. 4D): propodus with nine and eight clusters of setae on anteromedial margin and medial surface, respectively, palmar margin almost straight, with minutely serrate on the distal one third of the palmar margin. ***Oostegites*** on gnathopod 2 and pereopods 3–5, slender with long setae.

##### Variations.

***Mandible***: left and right incisors with two or three and three or four teeth, respectively; lacinia mobilis with four or five teeth. ***Gnathopod 2***: length of setae on coxa 0.4–0.9 × width of coxa. ***Pereopod 3***: length of setae on coxa 0.6–0.9 × width of coxa. ***Uropod 3***: the length-to-width ratio of inner ramus 1.5–2.5. ***Telson***: each lobe bearing two or three robust setae apically.

As body length increases, male gnathopod 2 shows morphological changes (Fig. 5A–C): in adult males (features of immature males in parentheses), propodus elongate (semioval), hump on palmar margin strongly produced (weakly produced), mid-palmar margin with long ridge (without ridge), posterior margin without robust seta (with a robust seta at distal end of posterior margin).

##### Coloration in life.

Eyes black; antennae 1 and 2 peduncular articles brown, flagellum articles have two black patterns on a white background; body laterally brown, dorsally black, with small white dot patterns on head, pereon, and abdomen. Males and females have similar body coloration.

##### Distribution.

South Korea: Busan (present study); Ulleungdo Island in Gyeongsangbuk-do (Kim and Kim 1991; Shin et al. 2025); Chujado Islands in Jeju-do (Kim et al. 2023). Japan: Chiba, Kanagawa, and Tottori; Shikinejima Island in Tokyo (present study) (Fig. 1).

##### Remarks.

Our specimens obtained from South Korea and Japan are well identical to the original description of *Elasmopus koreanus* by Kim and Kim (1991). A comparison of *E. koreanus* from Japan and South Korea showed no remarkable morphological differences other than their ontogenetic changes. The intraspecific genetic distances of COI between Japanese and Korean specimens of this species were at most 0.024 (*p*-distance) and 0.025 (K2P), respectively (Tables 3, 4). These values were not particularly large compared to the intraspecific genetic distances of the other *Elasmopus* species examined in this study (Tables 3, 4). Therefore, we concluded that *E. koreanus* from Japan and South Korea should be regarded as conspecific, since they show no significant morphological or genetic differences that would support their separation as distinct species.

**Table 3.** Uncorrected pairwise distances (*p*-distances) among *Elasmopus* species showing minimum–maximum values (mean value). “*n*” indicates the number of specimens, and “loc.” Indicates the number of collecting sites.

	*E. koreanus* (*n* = 3, loc. = 3)	*E. lumbiniger* sp. nov. (*n* = 6, loc. = 6)	*E. mukuinu* (*n* = 4, loc. = 4)	*E. nkjaf* (*n* = 2, loc. = 1)	*E. projectus* (*n* = 3, loc. = 2)	*E. rapax* (*n* = 6, loc. = 2)
*E. koreanus*	0.002–0.024 (0.016)					
*E. lumbiniger* sp. nov.	0.198–0.205 (0.201)	0.002–0.043 (0.017)				
*E. mukuinu*	0.202–0.207 (0.204)	0.167–0.184 (0.173)	0.003–0.050 (0.027)			
*E. nkjaf*	0.190–0.193 (0.192)	0.185–0.191 (0.188)	0.178–0.182 (0.180)	0.006 (-)		
*E. projectus*	0.178–0.182 (0.181)	0.158–0.170 (0.163)	0.164–0.176 (0.172)	0.187–0.191 (0.189)	0.008–0.024 (0.017)	
*E. rapax*	0.188–0.191 (0.190)	0.187–0.198 (0.190)	0.173–0.187 (0.178)	0.160–0.167 (0.162)	0.182–0.193 (0.186)	0.000–0.003 (0.001)

**Table 4.** Kimura two-parameter distances (K2P distances) among *Elasmopus* species showing minimum–maximum values (mean value). “*n*” indicates the number of specimens, and “loc.” Indicates the number of collecting sites.

	*E. koreanus* (*n* = 3, loc. = 3)	*E. lumbiniger* sp. nov. (*n* = 6, loc. = 6)	*E. mukuinu* (*n* = 4, loc. = 4)	*E. nkjaf* (*n* = 2, loc. = 1)	*E. projectus* (*n* = 3, loc. = 2)	*E. rapax* (*n* = 6, loc. = 2)
*E. koreanus*	0.002–0.025 (0.017)					
*E. lumbiniger* sp. nov.	0.233–0.244 (0.238)	0.002–0.044 (0.017)				
*E. mukuinu*	0.239–0.247 (0.243)	0.192–0.216 (0.201)	0.003–0.052 (0.028)			
*E. nkjaf*	0.222–0.226 (0.224)	0.215–0.222 (0.219)	0.205–0.211 (0.208)	0.006 (-)		
*E. projectus*	0.207–0.213 (0.210)	0.181–0.198 (0.189)	0.189–0.207 (0.200)	0.218–0.224 (0.221)	0.008–0.025 (0.018)	
*E. rapax*	0.217–0.224 (0.221)	0.219–0.235 (0.224)	0.200–0.219 (0.206)	0.211–0.226 (0.217)	0.186–0.196 (0.189)	0.000–0.003 (0.001)

*Elasmopus koreanus* is most similar to *E. mutatus* Barnard, 1962, with the following five features: 1) epimeral plate 3 posteroventral corner without distinct projection; 2) male gnathopod 2 propodus mid-palmar margin with subrectangular hump; 3) male gnathopod 2 propodus medial surface without dense setae; 4) pereopods 5–7 basis smooth, without long setae; and 5) telson broader than long. Kim and Kim (1991) distinguished *E. koreanus* from *E. mutatus* based on four morphological characters: 1) antennae of *E. koreanus* bear fewer setae than those of *E. mutatus*; 2) male gnathopod 2 palmar margin ridge where tip of dactylus contacts lacks a robust seta in *E. koreanus*, whereas it bears a robust seta in *E. mutatus*; 3) posterior margin of male gnathopod 2 propodus covered with fewer setae in *E. koreanus* than in *E. mutatus*; and 4) uropod 3 inner ramus broader in *E. koreanus* than in *E. mutatus*. However, all examined male specimens in the present study possessed a robust seta on the palm of the gnathopod 2 at the point where the tip of the dactylus contacts. This suggests that either Kim and Kim (1991) overlooked this robust seta, or that the presence or absence of this robust seta represents intraspecific variation in *E. koreanus*. Therefore, the presence or absence of this robust seta is not a diagnostic character distinguishing *E. koreanus* from *E. mutatus*. Additionally, regarding the broadness of the uropod 3 inner ramus, the length-to-width ratio in *E. koreanus* ranged from 1.5 to 2.5. In *E. mutatus*, this ratio was 2.1, which fell within the intraspecific variation range of *E. koreanus*. Thus, *E. koreanus* and *E. mutatus* cannot be distinguished based on the broadness of the uropod 3 inner ramus. On the other hand, we propose newly found taxonomic features to distinguish the two species: 1) antennae of *E. koreanus* bear fewer setae than those of *E. mutatus*; 2) posterior margin of male gnathopod 2 propodus covered with fewer setae in *E. koreanus* than in *E. mutatus*; and 3) the longest seta on coxae 2 and 3 shorter than the width of each coxa in *E. koreanus*, whereas longer in *E. mutatus*.

#### ﻿ Elasmopus lumbiniger sp. nov.

Taxon classificationAnimaliaAmphipodaMaeridae

A6AB1033-296A-5F52-9BB1-FAE054DAA466

https://zoobank.org/E0DF25E4-5009-4BC2-96C8-5434657629B2

**Figure 6.**
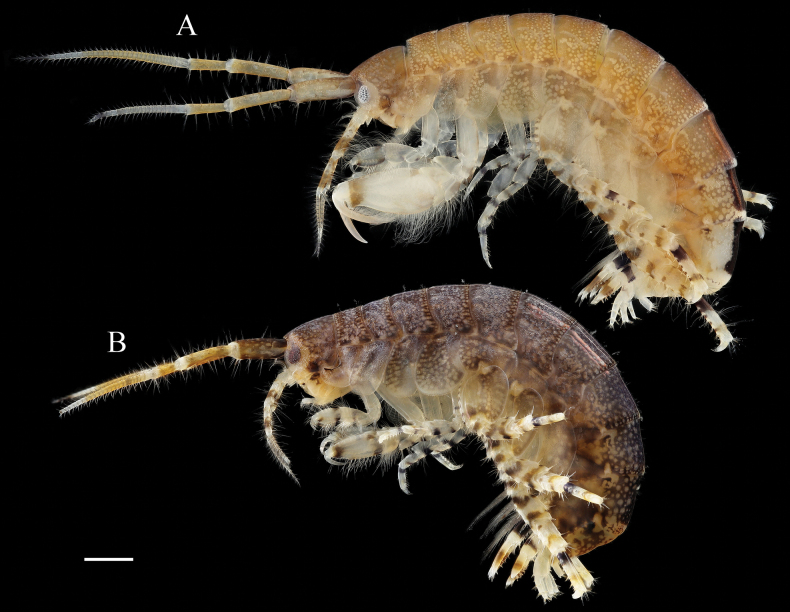
*Elasmopus lumbiniger* sp. nov. **A.** Paratype male, 13.8 mm (NSMT-Cr 33073), lateral view; **B.** Paratype female, 12.6 mm (NSMT-Cr 33075), lateral view. Scale bar: 1.0 mm.

**Figure 7.**
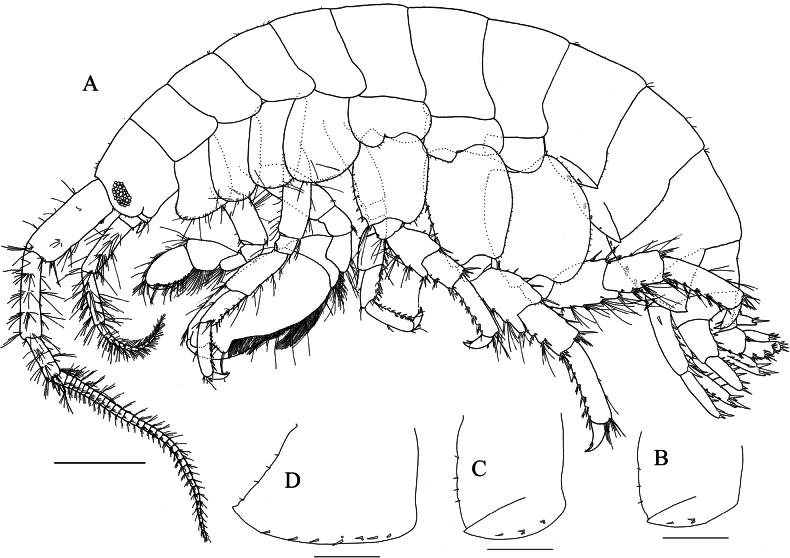
*Elasmopus lumbiniger* sp. nov. holotype male, 9.8 mm (NSMT-Cr 33064). **A.** Body, lateral view; **B.** Right epimeral plate 1, lateral view; **C.** Right epimeral plate 2, lateral view; **D.** Right epimeral plate 3, lateral view. Scale bars: 1.0 mm (**A**); 0.5 mm (**B–D**).

**Figure 8.**
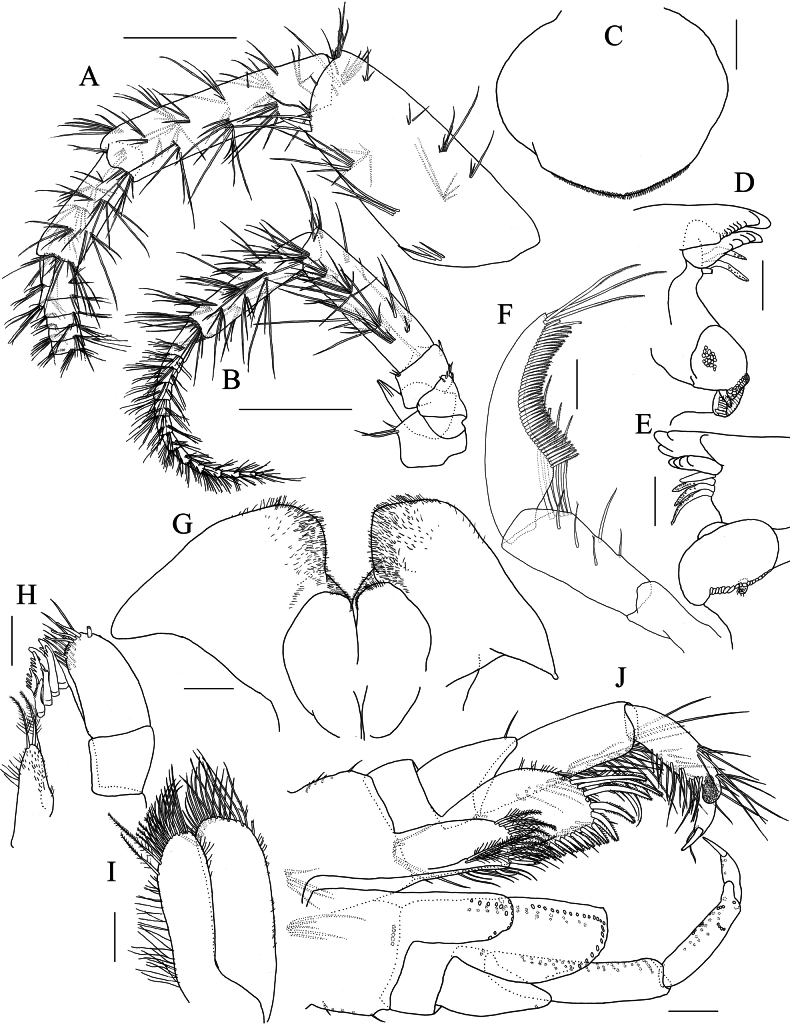
*Elasmopus lumbiniger* sp. nov. **A, B, D–J.** Holotype male, 9.8 mm (NSMT-Cr 33064); **C.** Paratype male, 9.9 mm (NSMT-Cr 33066). **A.** Right antenna 1, medial view; **B.** Left antenna 2, lateral view; **C.** Upper lip, anterior view; **D, E.** Incisor, lacinia mobilis, accessory setal row and molar process of left and right mandible, medial view; **F.** Left mandibular palp, medial view; **G.** Lower lip, ventral view; **H.** Right maxilla 1, dorsal view; **I.** Right maxilla 2, dorsal view; **J.** Maxilliped, dorsal view. Scale bars: 0.5 mm (**A, B**); 0.1 mm (**C–J**).

**Figure 9.**
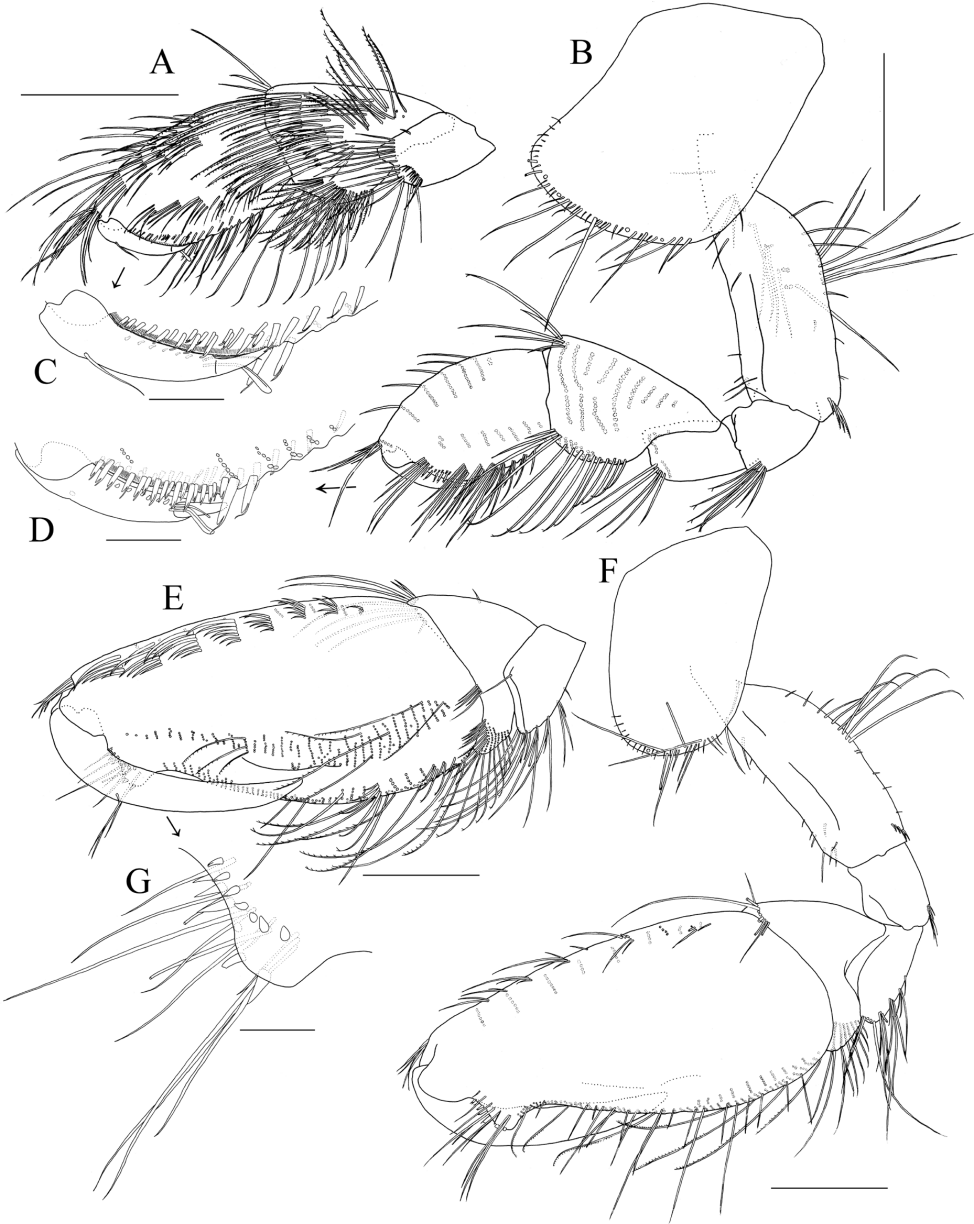
*Elasmopus lumbiniger* sp. nov. holotype male, 9.8 mm (NSMT-Cr 33064). **A.** Right gnathopod 1, medial view; **B.** Left gnathopod 1, lateral view; **C.** Palmar margin of right gnathopod 1 propodus, medial view; **D.** Palmar margin of left gnathopod 1 propodus, lateral view; **E.** Right gnathopod 2, medial view; **F.** Left gnathopod 2, lateral view, coxal gill omitted; **G.** Hump on right gnathopod 2 propodus, medial view. Gnathopod 2 coxal gill omitted. Scale bars: 0.5 mm (**A, B, E, F**); 0.1 mm (**C, D, G**).

**Figure 10.**
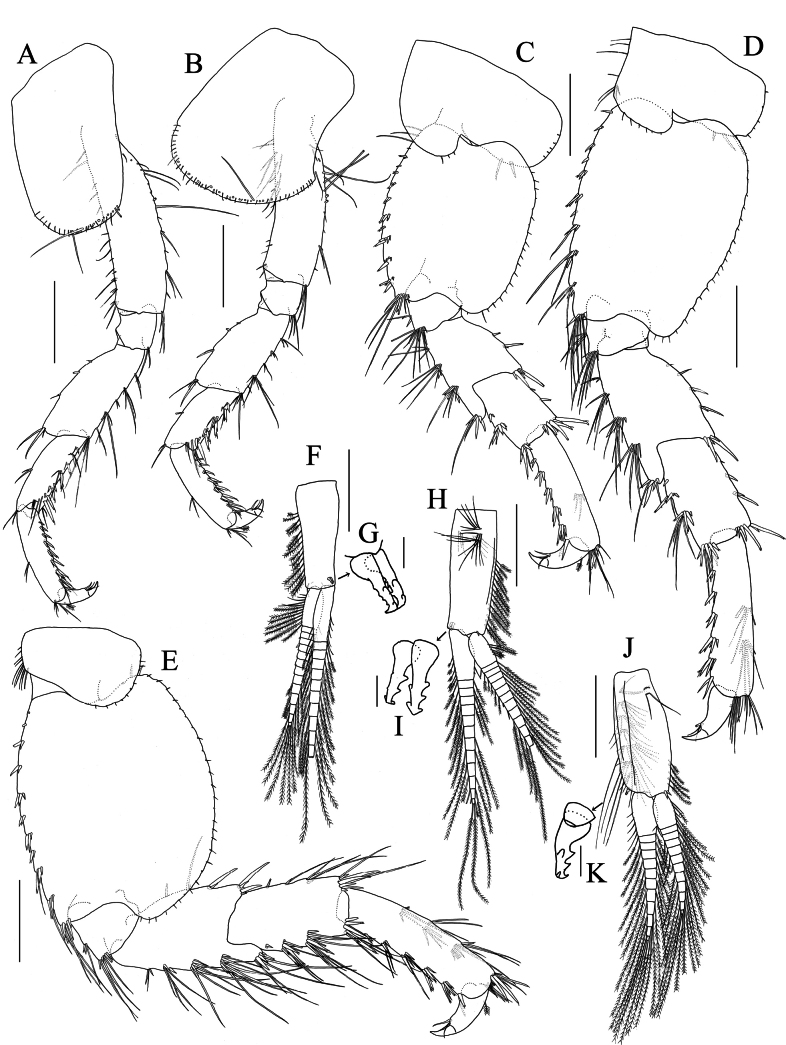
*Elasmopus lumbiniger* sp. nov. holotype male, 9.8 mm (NSMT-Cr 33064). **A–E.** Left pereopod 3–7, lateral view, coxal gills omitted; **F.** Left pleopod 1, posterior view; **G.** Retinacula of left pleopod 1, posterior view; **H.** Left pleopod 2, anterior view; **I.** Retinacula of left pleopod 2, anterior view; **J.** Left pleopod 3, anterior view; **K.** Retinacula of left pleopod 3, anterior view. Scale bars: 0.5 mm (**A–F, H, J**); 0.02 mm (**G, I, K**).

**Figure 11.**
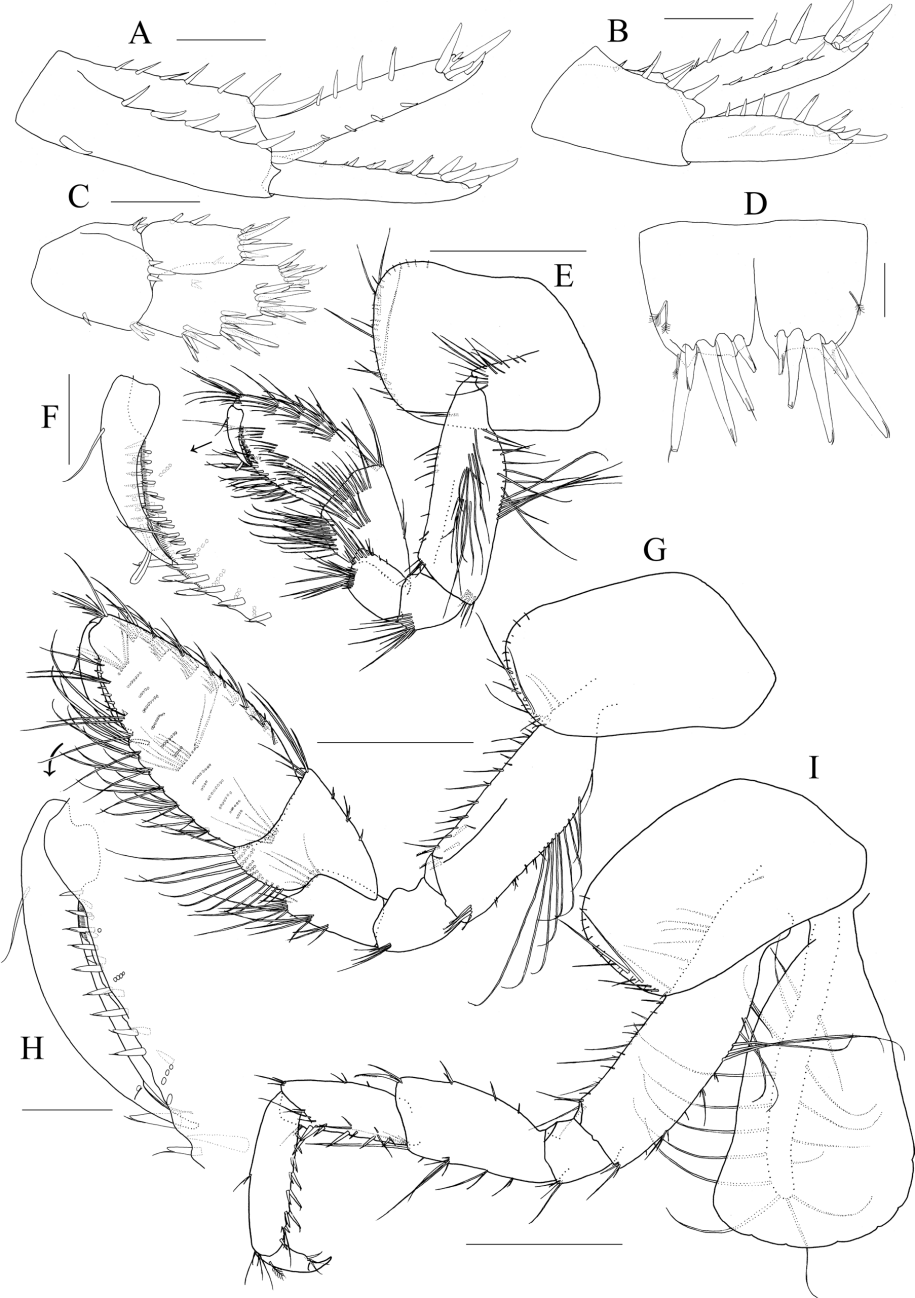
*Elasmopus lumbiniger* sp. nov. **A–D.** Holotype male, 9.8 mm (NSMT-Cr 33064); **E–I.** Paratype female, 8.7 mm (NSMT-Cr 33068). **A.** Left uropod 1, lateral view; **B.** Left uropod 2, lateral view; **C.** Right uropod 3, medial view; **D.** Telson, dorsal view; **E.** Right gnathopod 1, medial view; **F.** Palmar margin of right gnathopod 1 propodus, medial view; **G.** Left gnathopod2, lateral view, coxal gill and oostegite omitted; **H.** Palmar margin of left gnathopod 2 propodus, lateral view; **I.** Left pereopod 3, lateral view. Scale bars: 0.3 mm (**A–C**); 0.1 mm (**D, F, H**); 0.5 mm (**E, G, I**).

**Figure 12.**
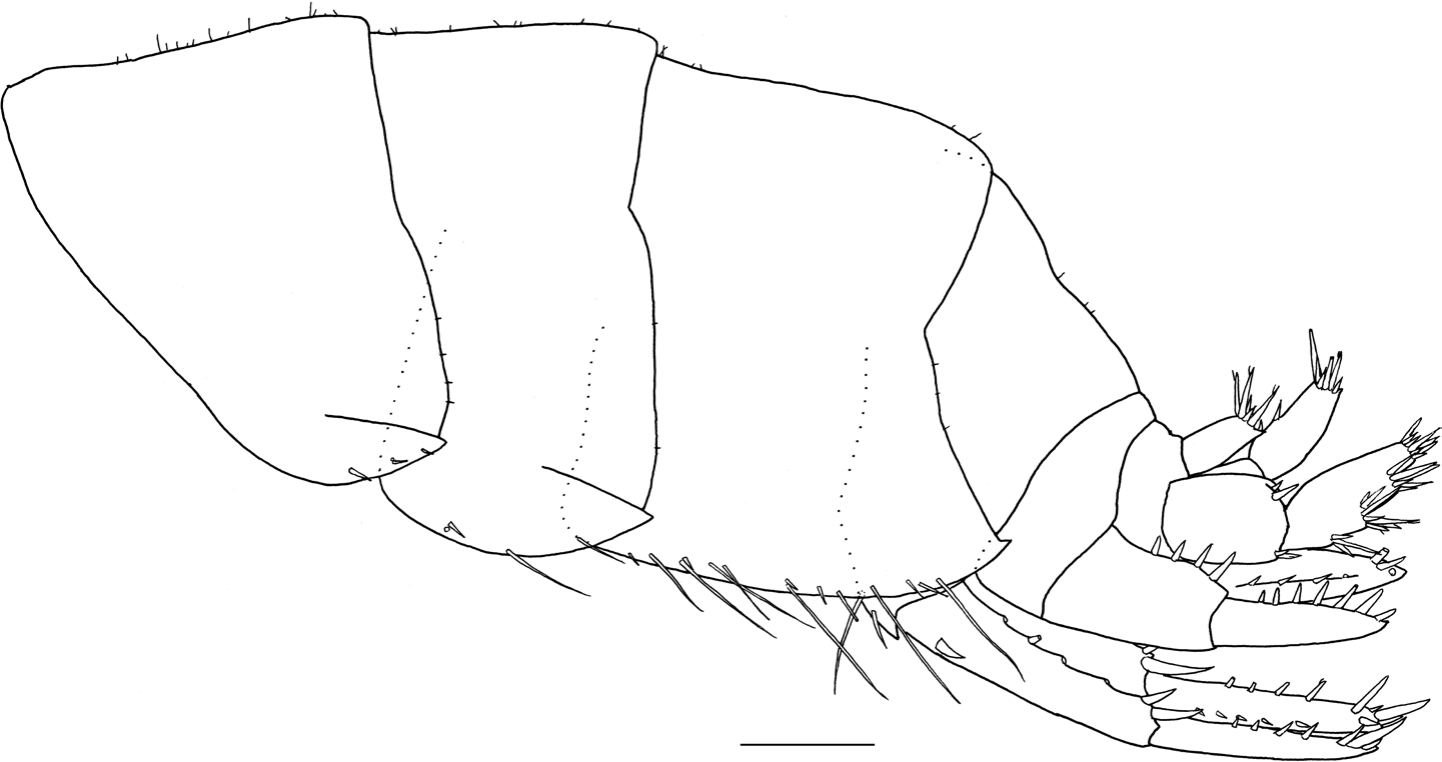
*Elasmopus lumbiniger* sp. nov., paratype male, 13.8 mm (NSMT-Cr 33073). Abdomen, lateral view. Scale bar: 0.5 mm.

Figs 6, 7, 8, 9, 10, 11, 12 [New Japanese name: Sewata-iso-yokoebi]

##### Type material.

***Holotype***: • male, 9.8 mm (NSMT-Cr 33064), Kannonzaki Coast, Yokosuka, Kanagawa, Japan (35.2592°N, 139.7436°E) (Fig. 1A), shallow subtidal zone, rocky shore, among holdfast of the brown alga *Eisenia bicyclis*, 4 February 2023, H. Yoshimura coll. ***Paratypes***: • male, 10.4, 9.9, 8.5 mm (NSMT-Cr 33065–33067), ovigerous female, 8.7 mm (NSMT-Cr 33068), same data as for the holotype; • male, 9.6, 7.1 mm (NSMT-Cr 33069, 33070), female, 5.9 mm (NSMT-Cr 33071), Kannonzaki Coast, Yokosuka, Kanagawa, Japan (35.2592°N, 139.7432°E) (Fig. 1A), shallow subtidal zone, rocky shore, among holdfast of the brown alga *Sargassum* sp., 22 April 2023, H. Yoshimura coll.; • male, 7.0 mm (NSMT-Cr 33072), Arasaki Coast, Yokosuka, Kanagawa, Japan (35.1947°N, 139.5996°E) (Fig. 1B), shallow subtidal zone, rocky shore, among the red alga *Corallina* sp., 2 September 2023, H. Yoshimura coll.; • male, 13.8 mm (NSMT-Cr 33073: Fig. 6A), Uchiura Beach, Kamogawa, Chiba, Japan (35.1262°N, 140.1893°E) (Fig. 1C), shallow subtidal zone, rocky shore, among the red alga *Corallina* sp., 18 June 2022, H. Yoshimura coll.; • male, 10.0 mm (NSMT-Cr 33074), Shirahama, Minamiboso, Chiba, Japan (34.9031°N, 139.8862°E) (Fig. 1D), intertidial zone, rocky shore, 5 June 2023, H. Ogawa coll.; • female, 12.6, 10.3 mm (NSMT-Cr 33075: Fig. 6B, NSMT-Cr 33076), Takurazaki, Wakayama, Wakayama, Japan (34.2671°N, 135.0605°E) (Fig. 1I), shallow subtidal zone, rocky shore, under the stone, 10 May 2024, H. Yoshimura coll.; • male, 10.0 mm (NSMT-Cr 33077), female, 7.9 mm (NSMT-Cr 33078), Uradome Beach, Iwami, Tottori, Japan (35.5921°N, 134.3198°E) (Fig. 1H), shallow subtidal zone, rocky shore, among sporophyll of the brown alga *Undaria pinnatifida* lying on the sea bottom, 16 June 2024, H. Ogawa, Y. Mukaida, M. Ooga, and H. Yoshimura coll.

##### Diagnosis.

Epimeral plate 3 posteroventral corner produced into well-developed acute spine. Antenna 1 peduncles bearing slender setae on lateral and medial margins, with tri-articulate accessory flagellum. Mandibular palp article 3 short. Gnathopod 2 carpus anterodistal corner with slender setae, length of the longest seta reaching 0.8–1.1 and 0.9–1.1 × width of carpus in male and female. Male gnathopod 2 propodus posterior margin and inner surface with dense slender setae, length of the longest seta reaching 0.9 × width of propodus, palmar margin with trapezoidal hump near hinge of dactylus, mid-palmer margin with ridge. Pereopods 5–7 basis posterior margin smooth, without long setae. Telson cleft, border than long; lobe trapezoid, with shallow incision at apex, each lobe bearing one plumose and 4–6 robust setae apically.

##### Description of male

**(holotype, NSMT-Cr 33064). *Body*** (Fig. 7A): smooth, not carinate, with a few short setae dorsally on pereon and abdomen. ***Head***: eyes oval; lateral cephalic lobe broad, anteroventral margin with notch; dorsal surface with a few short setae. ***Epimeral plates 1–3*** (Fig. 7B–D): each ventral margin with one cluster and one single robust seta, two clusters and one single robust seta, and two clusters and five robust setae, respectively; each posterior margin with three or four, four and four short setae, respectively; posteroventral corner of epimeral plates 1 and 2 weakly produced, epimeral plate 3 produced into well-developed acute spine.

***Antenna 1*** (Fig. 8A): length 0.55 × body length; peduncular articles 1–3 in length ratio of 1.1: 1.0: 0.6, with slender setae on lateral and medial margin; peduncular article 1 with three robust setae on posteroproximal margin, robust seta on posterodistal corner; accessory flagellum tri-articulate, accessory flagellar article 3 tiny; primary flagellum 34-articulate, with slender setae. ***Antenna 2*** (Fig. 8B): length 0.5 × antenna 1; peduncular article 2 with six short spines on anterodistal corner, gland cone of peduncular article 2 exceeding distal margin of peduncular article 3; peduncular article 4 slightly longer than 5, with slender setae on lateral and medial margins; flagellum 13-articulate, with slender setae.

***Mouthparts*.** As holotype upper lip missing, that of paratype (male, 9.9 mm, NSMT-Cr 33066) illustrated. ***Upper lip*** (Fig. 8C) with rounded anterior margin, bearing fine setae. ***Mandible*** (Fig. 8D–F) with left and right incisors two and four teeth, respectively; left lacinia mobilis five teeth and right lacinia mobilis four teeth; accessory setal row three setae on the left and four setae on the right; molar process well developed, triturative; palp (Fig. 8F) well developed, tri-articulate; palp article 1 without setae, article 2 with ten setae, article 3 falcate, length 3.0 × width. ***Lower lip*** (Fig. 8G) with outer lobes laterally expanded, bearing apical setae; inner lobes ovate, apically covered with setae. ***Maxilla 1*** (Fig. 8H) with inner plate bearing two plumose setae apically; outer plate with seven robust dentate setae; palp bi-articulate, article 1 marginally bare, article 2 with numerous apical and subapical setae, outer margin bare. ***Maxilla 2*** (Fig. 8I) with inner plate bearing long apical setae, with setae on outer and inner margins; outer plate bearing long apical setae, with setae on outer margin. ***Maxilliped*** (Fig. 8J) with inner plate bearing plumose setae apically; outer plate obovate, with spatula plumose setae on apical and inner margins, reaching more than half of palp article 2; palp article 1 with two setae on inner margin, with seta on outer margin; palp article 2 slender, lined with setae on inner margin, outer margin bare; nail of palp article 4 distinct.

***Gnathopod 1*** (Fig. 9A–D): subchelate; coxa anteroventral corner weakly produced, anterior margin weakly concave, ventral margin with long and short setae; basis with long setae on posterior margin and medial surface; carpus subequal in length to propodus, with slender setae on anterodistal corner, with dense pinnate sided setulated setae on medial surface and posterior margin; propodus with six clusters of setae on anterior margin, with seven clusters of setae on medial surface; palmar margin (Fig. 9C, D) almost transverse, minutely serrate, with rows of robust setae on medial and lateral palmar submargins, palmar corner with two long robust setae; dactylus with slender seta on anteroproximal margin, with six small setae on lateral margin, with spatulate seta and slender seta laterally at the base of nail. ***Gnathopod 2*** (Fig. 9E–G): subchelate; coxa with long and short setae on ventral margin, of which two long setae retrorse; basis with long setae on posterior margin; merus distoventral corner subquadrate bearing long setae; carpus with slender setae on anterodistal corner, length of these setae 0.9 × width of carpus, with dense setae on posterior margin; propodus with five clusters, two pairs and two single setae on anterolateral submargin, length of these setae 0.4 × width of propodus, anteromedial submargin with eight clusters of setae; lateral palmar margin weakly concave with trapezoidal hump (Fig. 9G) on near insertion of dactylus, with one clusters, three pairs and one single setae on lateral surface, with six and seven robust setae on lateral and medial margins, respectively; mid-palmar margin with ridge; posterior margin with row of plumose setae, the proximal seta longer than the distal seta, length of the longest seta 0.9 × width of propodus; dactylus strongly curved, subacute, length 0.6 × length of propodus, with seta on anteroproximal margin.

***Pereopod 3*** (Fig. 10A): coxa longer than broad, ventral margin with numerous short and a few long setae, of which long seta retrorse; posterior margins of basis with long slender and robust setae, merus with slender setae, carpus and propodus with robust and slender setae; dactylus with plumose seta on anteroproximal margin, with one robust and two slender setae at the base of nail. ***Pereopod 4*** (Fig. 10B): coxa expanded with posterior concavity, ventral margin arched bearing numerous short and a few long setae, of which long seta retrorse; posterior margins of basis with long slender and robust setae, merus with slender setae, carpus and propodus with robust and slender setae; dactylus with plumose seta on anteroproximal margin, with one robust and two slender setae at the base of nail. ***Pereopod 5*** (Fig. 10C): coxa bilobed, ventral margin of posterior lobe with two robust setae, posterior margin of posterior lobe with five short setae; basis broad, subrectangular, posterior margin smooth, with numerous short setae; anterior and posterior margins of carpus with robust setae; propodus with robust setae on anterior margin, with one cluster setae on posteromedial submargin; dactylus with plumose seta on posteroproximal margin, with one robust and two slender setae at the base of nail. ***Pereopod 6*** (Fig. 10D): coxa bilobed, anterior margin of anterior lobe with slender setae, ventral margin of posterior lobe with three robust setae, posterior margin of posterior lobe with five short setae; basis weakly expanded proximally, tapering distally, posterior margin smooth with numerous short setae; anterior margin of carpus with robust and slender setae, posterior margin with robust setae; anterior margin of propodus with robust setae, with two clusters setae on posteromedial submargin; dactylus with plumose seta on posteroproximal margin, with one robust seta and two slender setae at the base of nail. ***Pereopod 7*** (Fig. 10E): coxa rounded, with slender setae on anterior and short setae on posterior margins; basis expanded, subovate, posterior margin rounded, smooth, with numerous short setae; anterior and posterior margins of carpus with robust and slender setae; anterior margin of propodus with robust and slender setae, with three clusters setae on posteromedial submargin; dactylus with plumose seta on posteroproximal margin, with one robust and two slender setae at the base of nail.

Coxal gills on gnathopod 2 and pereopods 3–6.

***Pleopods*** (Fig. 10F–K): peduncles with long setae, inner distal corner of peduncle with two retinacula (Fig. 10G, I, K), peduncle of pleopod 3 broader than pleopod 1 and 2.

***Uropods*. *Uropod 1*** (Fig. 11A): peduncle length 1.2 × length of outer ramus, with six dorsolateral, seven dorsomedial and one basofacial robust setae; outer ramus slightly shorter than inner ramus, medial and lateral margins each with four robust setae, distal part with four robust setae; inner ramus medial and lateral margins each with four robust setae, distal part with five robust setae. ***Uropod 2*** (Fig. 11B): peduncle length 0.9 × length of outer ramus; outer ramus slightly shorter than inner ramus, with three and five robust setae on medial and lateral margins, respectively, distal part with four robust setae; inner ramus five and four robust setae on medial and lateral margins, respectively, distal part with five robust setae. ***Uropod 3*** (Fig. 11C): peduncle length 0.8 × length of outer ramus, with pair of robust setae on outer margin; rami distally truncated; outer ramus length 1.5 × length of inner ramus, with three and one clusters of robust setae on lateral and distal margins, respectively, with robust seta and one pair of robust setae on medial margin and surface, respectively; inner ramus with cluster of robust setae distally and two robust setae on medial margin.

***Telson*** (Fig. 11D): broader than long, cleft for 75% of length; lobe trapezoid, with shallow incision at apex, each lobe bearing one plumose five robust setae apically, lateral margin of each lobe with one or two plumose setae.

##### Description of female

**(paratype, NSMT-Cr 33068), sexually dimorphic characters. *Gnathopod 1*** (Fig. 11E, F): propodus anterior margin with 1 single and 4 clusters of setae, medial surface with 6 clusters of setae. ***Gnathopod 2*** (Fig. 11G, H): slender setae on carpus anterodistal corner reaching width of carpus; propodus with two clusters, three pairs, and two2 single setae on anterolateral submargin, length of these setae 0.7 × width of propodus, anteromedial submargin with 13 clusters; palmar margin (Fig. 11H) almost straight, with minutely serrate on the distal 1/5 of the palmar margin. ***Pereopod 3*** (Fig. 11I): basis with slender setae on anterior margin, the longest seta 0.8 × width of basis. ***Oostegites*** on gnathopod 2 and pereopods 3–5, slender with long setae.

##### Variations.

***Mandible***: left and right incisors with two or three and three or four teeth, respectively; lacinia mobilis with four or five teeth. ***Gnathopod 2***: length of setae on carpus anterodistal corner 0.8–1.1 and 0.9–1.1 × width of carpus in male and female, length of setae on propodus anterolateral submargin 0.3–0.7 and 0.6–0.7 × width of propodus in male and female, respectively. ***Telson***: each lobe bearing 4–6 robust setae apically.

Large male > 10 mm bearing slender setae on ventral margins of epimeral plates 2 and 3, and urosomite 1 (Fig. 12).

##### Coloration in life.

Male: eyes black; antenna 1 peduncular articles brown, flagellum with two patterns, brown proximally and black distally, on a white background; body color varies among individuals ranging from reddish brown to brown or black, with small white dot patterns on head, pereon, abdomen, and coxae, with one dark black band along dorsal midline of pleonite 3 and urosomite 1. Female: body black, without distinct black band on dorsal midline of abdomen.

##### Distribution.

Japan: Chiba, Kanagawa, Wakayama, and Tottori (present study) (Fig. 1).

##### Ecological note.

This new species was found among brown and red algae or under stones in rocky shore. *E. lumbiniger* sp. nov. and *E. projectus* were collected from the same individual algae holdfasts at two sites: Kannonzaki Coast (Fig. 1A) and Uradome Beach (Fig. 1H).

##### Etymology.

The specific name *lumbiniger*, from the Latin *lumbus* (= loin, lower back) and *niger* (= black), refers to the dark coloration along the dorsal midline of male pleonite 3 and urosomite 1 in life (Fig. 6A). The Japanese name is derived from this band, which resembles a vein running along the back of a shrimp, known as “Sewata”.

##### Remarks.

*Elasmopus lumbiniger* sp. nov. is morphologically similar to *E. antennatus* (Stout, 1913), *E. balkomanus* Thomas & Barnard, 1988, and *E. lemaitrei* Ortiz & Lalana, 1994, in having the following features: 1) epimeral plate 3 posteroventral corner produced into well-developed acute spine; 2) male gnathopod 2 propodus palm with dense slender setae; 3) male gnathopod 2 mid-palmar margin with ridge; 4) male gnathopod 2 propodus with hump on near insertion of dactylus; 5) pereopods 5–7 basis posterior margin smooth, without long setae; and 6) telson broader than long. However, *E. lumbiniger* sp. nov. can be distinguished from the above three species by length of the longest seta on the anterodistal corner of male gnathopod 2 carpus 0.8–1.1 × width of carpus (less than 0.2 in these three species). From *E. antennatus*, this new species is distinguishable by the following two characters in addition (features of *E. antennatus* in parentheses): 1) hump on propodus of male gnathopod 2 trapezoidal, produced (semioval, weekly produced); and 2) the length of setae on anterior margin of pereopods 5–7 merus exceeding half the width of each merus (less than half of the width of each merus). *Elasmopus lumbiniger* sp. nov. can be further distinguished from *E. balkomanus* and *E. lemaitrei* by the following two additional features (features of *E. balkomanus* and *E. lemaitrei* in parentheses): 1) ridge on mid-palmar margin of male gnathopod 2 propodus not serrate (serrate); and 2) the length of setae on posterior margin of pereopod 7 carpus almost equal to that on anterior margin (more than twice that on anterior margin).

In this study, we calculated the genetic distances of COI among five *Elasmopus* species recorded in East Asia and *Elasmopus lumbiniger* sp. nov. *E. lumbiniger* sp. nov. differs from the other five *Elasmopus* species by large genetic distances (0.158–0.205 in *p*-distance; 0.181–0.244 in K2P), which were larger than the intraspecific distances among the *Elasmopus* species examined in this study (Tables 3, 4). In addition, *E. lumbiniger* sp. nov. is morphologically distinguished from its congeners. Therefore, it can be concluded that *E. lumbiniger* sp. nov. is a distinct new species.

#### ﻿ Elasmopus mukuinu

Taxon classificationAnimaliaAmphipodaMaeridae

Sir & White, 2022

967A4C8D-AA6D-5A26-9D40-336937981218

**Figure 13.**
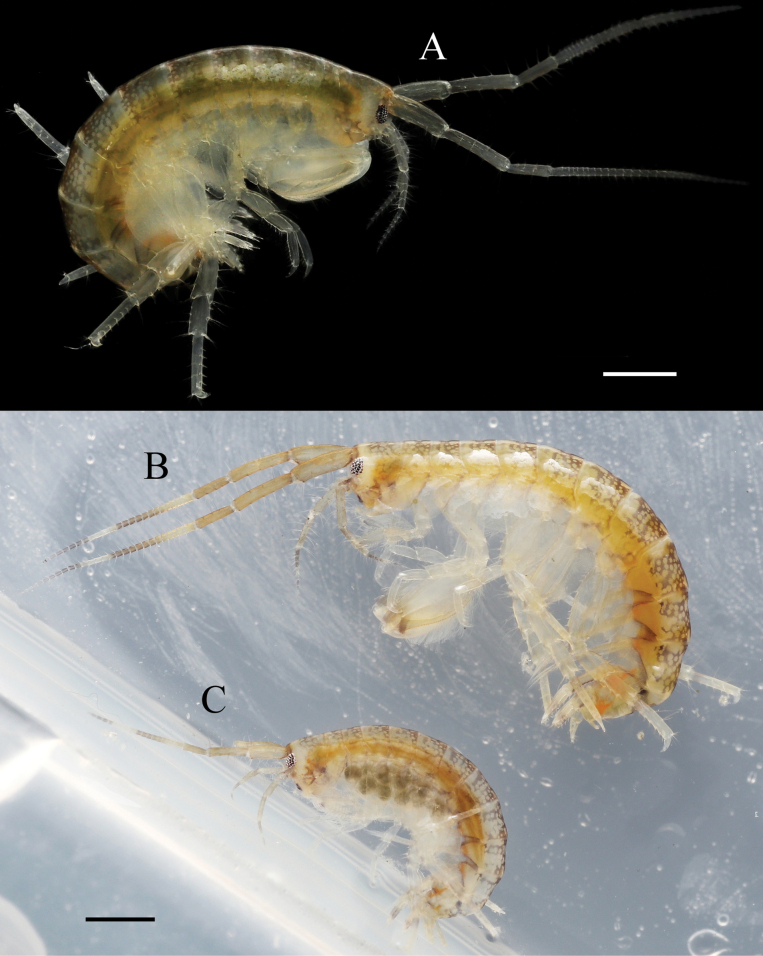
*Elasmopus mukuinu*. **A.** Male, 9.3 mm (NSMT-Cr 33079), lateral view; **B.** Male, 8.7 mm (NSMT-Cr 33081), lateral view; **C.** Female, 5.1 mm (NSMT-Cr 33083), lateral view. Scale bars: 1.0 mm.

**Figure 14.**
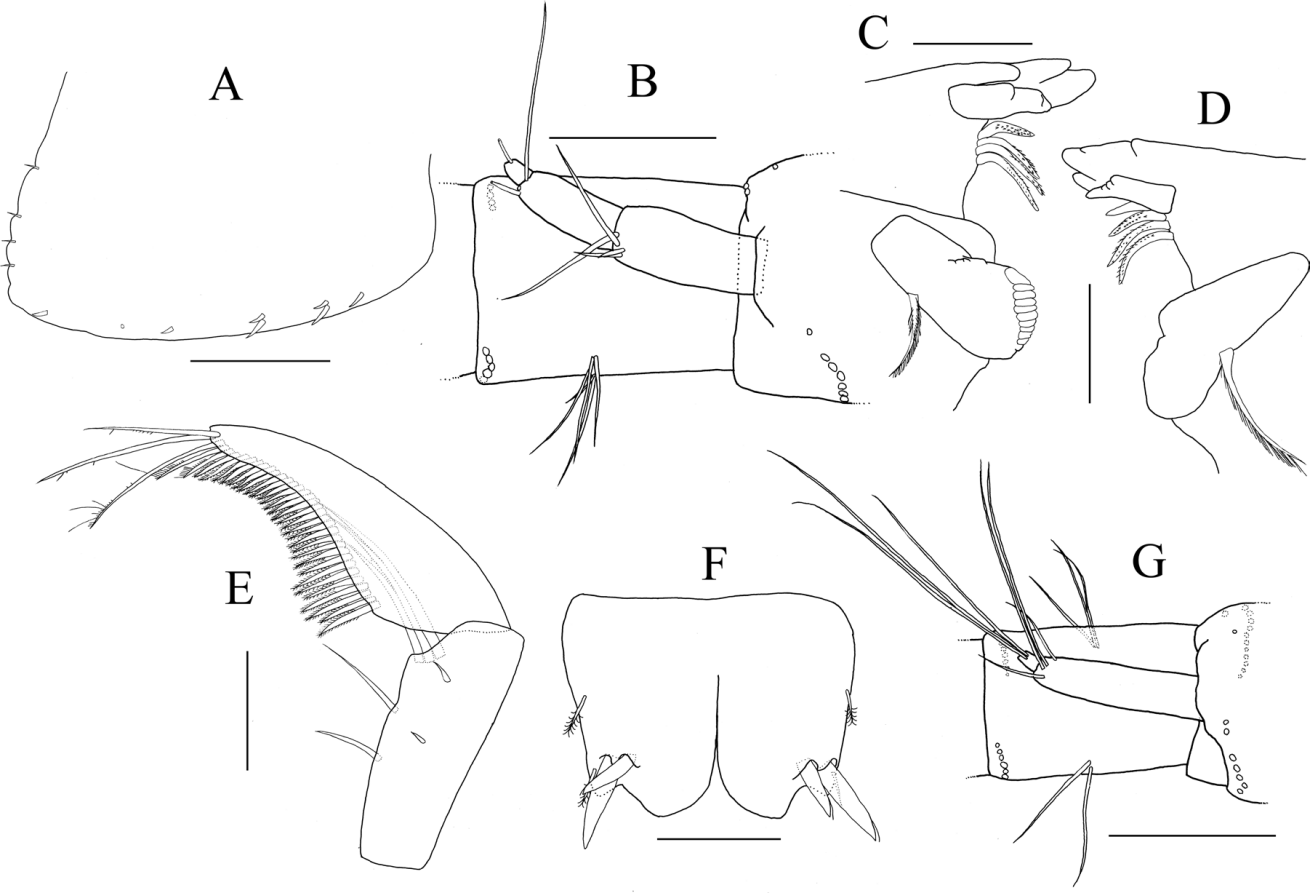
*Elasmopus mukuinu*. **A–F.** Male, 8.7 mm (NSMT-Cr 33081); **G.** Female, 5.1 mm (NSMT-Cr 33083). **A.** Right epimeral plate 3, lateral view; **B.** Right accessory flagellum, medial view; **C, D.** Incisor, lacinia mobilis, accessory setal row and molar process of left and right mandible, medial view; **E.** Right mandibular palp articles 2, 3, medial view; **F.** Telson, dorsal view; **G.** Right accessory flagellum, medial view. Scale bars: 0.3 mm (**A**); 0.1 mm (**B–G**).

**Figure 15.**
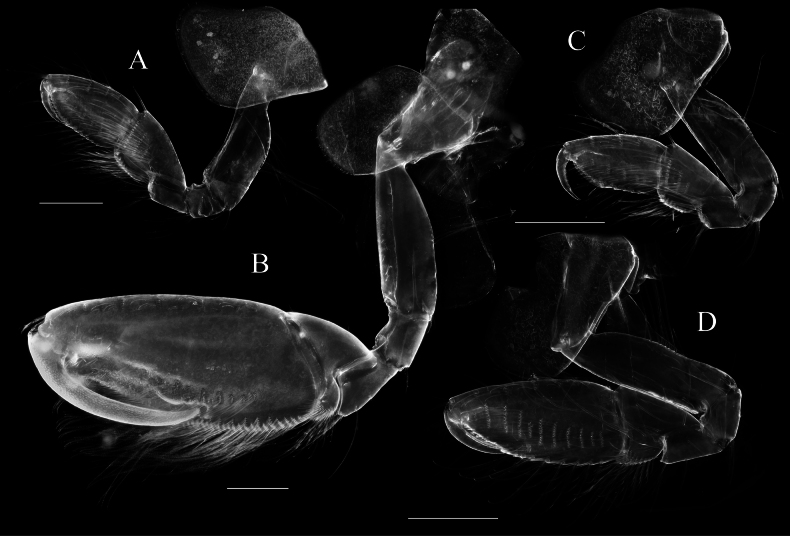
*Elasmopus mukuinu*. **A, B.** Male, 8.7 mm (NSMT-Cr 33081); **C, D.** Female, 5.1 mm (NSMT-Cr 33083). **A.** Right gnathopod 1, medial view; **B.** Right gnathopod 2, medial view; **C.** Right gnathopod 1, medial view; **D.** Right gnathopod 2, medial view. Scale bars: 0.3 mm.

Figs 13, 14, 15 [New Japanese name: Mukuinu-iso-yokoebi]

Elasmopus mukuinu Sir & White, 2022: 574, figs 1C, D, 4–9.

##### Material examined.

• Male, 9.3 mm (NSMT-Cr 33079: Fig. 13A), Kaichu Doro, Uruma, Okinawa, Japan (26.3324°N, 127.9222°E) (Fig. 1J), intertidal zone, coral beach, among the green alga *Ulva* sp., 14 March 2025, H. Yoshimura coll.; • ovigerous female, 7.5 mm (NSMT-Cr 33080), Ono Beach, Shimoda, Shizuoka, Japan (34.6329°N, 138.8975°E) (Fig. 1E), shallow subtidal zone, rocky shore, under the stone, 24 February 2024, H. Yoshimura coll.; • male, 8.7, 7.4 mm (NSMT-Cr 33081: Fig. 13B, NSMT-Cr 33082), ovigerous female, 5.1 mm (NSMT-Cr 33083: Fig. 13C), Oura Beach, Shikinejima, Tokyo, Japan (34.3307°N, 139.2083°E) (Fig. 1F), shallow subtidal zone, rocky shore, under the stone, 3 August 2024, H. Yoshimura coll.; • male, 14.6, 7.9, 6.5 mm (NSMT-Cr 33084–33086), Yakengahama Beach, Hachijojima, Tokyo, Japan (33.1007°N, 139.7696°E) (Fig. 1G), rocky shore, 20 December 2022, K. Tomikawa coll.

##### Type locality.

Kaichu Doro, Okinawa Island, Japan (26.3321°N, 127.9146°E).

##### Diagnosis.

Epimeral plate 3 posterior margin weakly serrated. Antenna 1 peduncles bearing slender setae on lateral and medial margins, with bi- or tri-articulate accessory flagellum. Mandibular palp article 3 short. Gnathopod 2 carpus anterodistal corner with slender setae, length of the longest seta reaching 0.3–0.6 and 0.4–0.6 × width of carpus in male and female. Male gnathopod 2 propodus posterior margin and inner surface with dense slender setae, length of the longest seta 0.9 × width of propodus, palmar margin with semicircular hump near hinge of dactylus, mid-palmer margin with ridge. Pereopods 5–7 basis posterior margin smooth, without long setae. Telson cleft, border than long; lobe trapezoid, with shallow incision at apex, inner apical corner rounded and produced, each lobe bearing one plumose and one or two robust setae apically.

##### Description of male

**(NSMT-Cr 33081). *Body*** (Fig. 13B): smooth, not carinate, with a few short setae dorsally on pereon and abdomen. ***Head***: eyes oval; lateral cephalic lobe broad, anteroventral margin with notch; dorsal surface with a few short setae. ***Epimeral plate 3*** (Fig. 14A): ventral margin with two pairs and four single robust setae, posterior margin weakly serrated, with four short setae.

***Antenna 1***: length 0.6 × body length; peduncular articles 1–3 with slender setae on lateral and medial margins; accessory flagellum (Fig. 14B) tri-articulate, reaching to distal margin of primary flagellar article 1, accessory flagellar article 3 tiny; primary flagellum 28-articulate, with slender setae.

***Mandible*** (Fig. 14C–E) with left and right incisors three and four teeth, respectively; left and right lacinia mobilis three and four teeth, respectively; accessory setal row consisting of four setae on each of left and right mandibles; molar process well developed, triturative; palp well developed, tri-articulate; article 2 with seven setae, article 3 falcate, length 3.2 × width.

***Gnathopod 1*** (Fig. 15A): subchelate; coxa anteroventral corner weakly produced; basis with long setae on posterior margin and medial surface; carpus subequal in length to propodus, with slender seta on anterodistal corner, with dense setae on posterior margin and medial surface; propodus with four and five clusters of setae on anterior margin and medial surface, respectively, posterior margin with row of slender and robust setae, palmar margin almost transverse, minutely serrate, with rows of robust setae on medial and lateral palmar submargins. ***Gnathopod 2*** (Fig. 15B): subchelate; basis with long setae on posterior margin; carpus with slender setae on anterodistal corner, length of these setae reaching 0.6 × width of carpus, with dense setae on posterior margin; propodus with one cluster, one pair and three single setae on anterolateral submargin, length of these setae reaching 0.2 × width of propodus, with nine clusters of setae on anteromedial submargin, lateral palmar margin with hump on near insertion of dactylus, mid-palmar margin with ridge, posterior margin with row of plumose setae, the proximal seta longer than the distal seta, length of the longest seta reaching 0.9 × width of propodus.

***Telson*** (Fig. 13F): broader than long, cleft for 65% of length; lobe trapezoid, with shallow incision at apex, inner apical corner rounded and produced, each lobe bearing one plumose and two robust setae apically, lateral margin of each lobe with one plumose seta.

##### Description of female

**(NSMT-Cr 33083), sexually dimorphic characters. *Antenna 1***: accessory flagellum (Fig. 14G) bi-articulate. ***Gnathopod 1*** (Fig. 15C): propodus anterior margin with three clusters of setae, medial surface with five clusters of setae. ***Gnathopod 2*** (Fig. 15D): slender setae on carpus anterodistal corner 0.6 × width of carpus; propodus with four slender setae on anterolateral submargin, the longest setae 0.4 × width of propodus, with eight and ten clusters of setae on anteromedial margin and medial surface, respectively; palmar margin almost straight, with minutely serrate on the distal 2/5 of the palmar margin.

##### Variations.

***Mandible***: both incisors with 2–4 teeth; right lacinia mobilis with four or five teeth. ***Gnathopod 2***: length of setae on carpus anterodistal corner 0.3–0.6 and 0.4–0.6 × width of carpus in male and female, length of setae on propodus anterolateral submargin 0.2–0.3 and 0.3–0.5 × width of propodus in male and female, respectively. ***Telson***: each lobe bearing one or two robust setae apically.

##### Coloration in life.

Eyes black; antennae 1 and 2 peduncular articles brown, flagellum articles have two black patterns on a white background; body translucent white, laterally brown, with small white dot patterns on head, pereon, and abdomen, with orange pattern on epimeral plates. Male and female have similar body coloration.

##### Distribution.

Japan: Shizuoka, Izu Islands (Shikinejima Island and Hachijojima Island), Tokyo (present study); Okinawa Island, Okinawa (Sir and White 2022; present study) (Fig. 1).

##### Remarks.

The examined specimens agreed well with the original description of *E. mukuinu* by Sir and White (2022), but there was regional morphological variation in the number of accessory flagellum articles of antenna 1. In the male from Okinawa Island (Fig. 1J) and females from Shizuoka (Fig. 1E) and Shikinejima Island (Fig. 1F), the accessory flagellum consisted of two articles, consistent with the original description (like Fig. 14G). In contrast, males from Shikinejima Island and Hachijojima Island (Fig. 1G) possessed a tri-articulate accessory flagellum (like Fig. 14B). Therefore, the number of accessory flagellum articles in males differed between localities: *E. mukuinu* from Okinawa Island had two articles, whereas those from the Izu Islands (Shikinejima and Hachijojima Islands) had three.

The genetic distance of COI between *E. mukuinu* from Okinawa Island and those from Shizuoka and the Izu Islands was 0.050 (*p*-distance) and 0.052 (K2P), respectively, representing the largest intraspecific genetic distance among the *Elasmopus* species examined in this study (Tables 3, 4). However, we believed that these morphological and genetic differences were not sufficient to support separation into distinct species, and we concluded that these specimens should be regarded as conspecific. Further examination of *E. mukuinu* specimens from other regions will help clarify the taxonomic status of local populations of this species.

### ﻿Genetic distances among *Elasmopus* species

The intra- and interspecific distances in *Elasmopus* were calculated using *p*-distance (Table 3) and K2P distance (Table 4). The maximum intraspecific distances were 0.050 (*p*-distance) and 0.052 (K2P), and the minimum interspecific distances were 0.158 (*p*-distance) and 0.181 (K2P).

### ﻿Key to the species of *Elasmopus* from East Asia

The following dichotomous key excludes *E. spinimanus*, whose occurrence in East Asia is doubtful.

**Table:** 

1	Urosomite 1 with triangular process dorsally	***E. japonicus***
–	Urosomite 1 dorsally smooth	**2**
2	Male gnathopod 2 propodus medial surface and palmar margin with few setae	**3**
–	Male gnathopod 2 propodus medial surface and palmar margin with dense slender setae	**6**
3	Antenna 2 flagellum articles anteroposteriorly compressed; pereopods 5–7 bases without long setae on posterior margins	***E. koreanus***
–	Antenna 2 flagellum articles nearly cylindrical; pereopods 5–7 bases with long setae on posterior margins	**4**
4	Telson shorter than wide	***E. hooheno***
–	Telson longer than wide	**5**
5	Foremost medial tooth on palm of male gnathopod 2 not exceeding palmar margin; posterodistal setae on propodi of pereopods 5–7 longer than length of dactyli	***E. rapax***
–	Foremost medial tooth on palm of male gnathopod 2 exceeding palmar margin; posterodistal setae on propodi of pereopods 5–7 shorter than length of dactyli	***E. nkjaf***
6	Posterior margin of pereopod 6 basis castelloserrate	**7**
–	Posterior margin of pereopod 6 basis smooth	**8**
7	Posterior margin of pereopod 7 basis castelloserrate; epimeral plate 3 posteroventral corner with distinct spine	***E. nanshaensis***
–	Posterior margin of pereopod 7 basis smooth; epimeral plate 3 posteroventral corner notched, without distinct spine	***E. alkhiranensis***
8	Inner margins of pereopods 3–7 dactyli serrate	***E. spinidactylus***
–	Inner margins of pereopods 3–7 dactyli smooth	**9**
9	Male gnathopod 2 carpus with a row of slender setae along anterior margin	***E. spinicarpus***
–	Male gnathopod 2 carpus anterior margin with setae on anterodistal corner only, or on both anterodistal corner and mid-anterior margin	**10**
10	Anterior setae on meri of pereopods 5–7 shorter than half width of meri	***E. hawaiensis* sensu Ren, 2012**
–	Anterior setae on meri of pereopods 5–7 longer than half width of meri	**11**
11	Male gnathopod 2 propodus mid-palmer margin with well-developed process; length of setae on gnathopod 2 carpus anterodistal corner less than 0.2 × width of carpus in male, less than 0.4 × in female; male pereopod 7 merus to propodus without slender setae on posterior margins	***E. projectus***
–	Male gnathopod 2 propodus mid-palmer margin with ridge; length of setae on gnathopod 2 carpus anterodistal corner exceeding 0.3 × width of carpus in male, exceeding 0.4 × in female; male pereopod 7 merus to propodus with slender setae on posterior margins	**12**
12	Epimeral plate 3 posteroventral corner without distinct projection; length of setae on gnathopod 2 carpus anterodistal corner 0.3–0.6 and 0.4–0.6 × width of carpus in male and female; telson each lobe bearing 1 or 2 robust setae apically	***E. mukuinu***
–	Epimeral plate 3 posteroventral corner produced into well-developed acute spine; length of setae on gnathopod 2 carpus anterodistal corner 0.8–1.1 and 0.9–1.1 × width of carpus in male and female; telson each lobe bearing 5 or 6 robust setae apically	***E. lumbiniger* sp. nov.**

## ﻿Discussion

### ﻿Species diversity of *Elasmopus* in East Asia

To date, thirteen species of *Elasmopus* have been recorded in East Asia. In the southern limit of this region, eight species are known from South China Sea (Table 1; Ren 2012; Myers and Momtazi 2015): *E. alkhiranensis*, *E. hawaiensis*, *E. hooheno*, *E. nanshaensis*, *E. rapax*, *E. spinicarpus*, *E. spinidactylus*, and *E. spinimanus*. Among these, the record of *E. spinimanus* by Ren (2012) was based solely on a single female specimen. This species was originally described from Sri Lanka based on mainly male specimens (Walker 1904). The female morphological characters of *E. spinimanus* were later described from specimens collected in Fiji (Myers 1985). We compared the female morphology of *E. spinimanus* described by Ren (2012) and Myers (1985) and found that they closely agreed. However, accurate species identification within *Elasmopus* requires examination of male morphological futures. Therefore, the identification of *E. spinimanus* by Ren (2012) is doubtful. Further taxonomic investigation, including examination of male specimens from the same population of *E. spinimanus* sensu Ren (2012), is necessary to confirm its taxonomic position.

At its northern limit, *E. smirnovi* Bulyčeva, 1952 was described from Primorsky Krai, Russian Far East (Bulyčeva 1952). However, this species exhibits morphological characteristics that differ from those of *Elasmopus* and is suggested to belong to the *Maera* group (Vader and Krapp-Schickel 2012). Because *E. smirnovi* could not belong to *Elasmopus*, Japanese and South Korean waters represent the true northernmost distribution of this genus in the northwestern Pacific and are important for understanding its diversity. Until now, only two species, *E. japonicus* and *E. projectus*, had been recorded in temperate regions of Japan, and two species, *E. koreanus* and *E. rapax*, from South Korea. However, the present study recorded three additional species from temperate Japan: *E. koreanus*, *E. lumbiniger* sp. nov., and *E. mukuinu*. These findings suggest that the species diversity of *Elasmopus* in northern East Asia is higher than previously recognized. Further surveys in previously unstudied regions of Japan and South Korea could find additional unrecorded or undescribed species.

## ﻿Conclusions

*Elasmopus lumbiniger* sp. nov., found in Japan, is distinguished from its congeners by its morphological features. *Elasmopus koreanus*, previously known only in Korea, is newly recorded in Japan. *Elasmopus mukuinu*, which has been reported only from Okinawa Island, is also found along the temperate coast of Japan, greatly extending its known distribution range. These findings shed light on the high species diversity of *Elasmopus* in waters around Japan and South Korea. Furthermore, the nucleotide sequences of the COI of these species are provided.

## Supplementary Material

XML Treatment for Elasmopus koreanus

XML Treatment for Elasmopus lumbiniger

XML Treatment for Elasmopus mukuinu

## ﻿Additional information

### Use of AI

No use of AI was reported.

### Author ORCIDs

Hiro Yoshimura https://orcid.org/0009-0005-1297-4368

Chi-Woo Lee https://orcid.org/0000-0002-0763-4271

Ko Tomikawa https://orcid.org/0000-0003-1521-9016

### Data availability

All of the data that support the findings of this study are available in the main text or Supplementary Information.
